# Simulation and annotation of global acronyms

**DOI:** 10.1093/bioinformatics/btac298

**Published:** 2022-04-28

**Authors:** Maxim Filimonov, Daphné Chopard, Irena Spasić

**Affiliations:** School of Computer Science and Informatics, Cardiff University, Cardiff CF24 4AG, UK; School of Computer Science and Informatics, Cardiff University, Cardiff CF24 4AG, UK; School of Computer Science and Informatics, Cardiff University, Cardiff CF24 4AG, UK

## Abstract

**Motivation:**

Global acronyms are used in written text without their formal definitions. This makes it difficult to automatically interpret their sense as acronyms tend to be ambiguous. Supervised machine learning approaches to sense disambiguation require large training datasets. In clinical applications, large datasets are difficult to obtain due to patient privacy. Manual data annotation creates an additional bottleneck.

**Results:**

We proposed an approach to automatically modifying scientific abstracts to (i) simulate global acronym usage and (ii) annotate their senses without the need for external sources or manual intervention. We implemented it as a web-based application, which can create large datasets that in turn can be used to train supervised approaches to word sense disambiguation of biomedical acronyms.

**Availability and implementation:**

The datasets will be generated on demand based on a user query and will be downloadable from https://datainnovation.cardiff.ac.uk/acronyms/.

## 1 Introduction

Acronyms are systematic abbreviations of frequently mentioned words and phrases. Their formation follows special capitalization and blending patterns (Fandrych, 2008). They are introduced primarily to support efficiency of written communication in terms of time and space. From the reading perspective, familiarity (prior experience processing a given stimulus), rather than orthographic regularity, plays a critical role in rapidly translating an acronym from percept to meaning (Laszlo and Federmeier, 2007). Therefore, the use of globally accepted acronyms, which are commonly used as synonyms of prominent domain-specific concepts, e.g. DNA (deoxyribonucleic acid), should pose no major difficulties in specialist communication between domain experts. Indeed, clinical narratives feature extensive use of acronyms, which, unlike their counterparts in formal scientific writing, are not defined explicitly in documents that refer to them. However, when the content of such documents needs to be analyzed automatically, their use can hinder the performance of natural language processing (NLP) algorithms (Moon *et al.*, 2014; Spasić *et al.*, 2019). For example, when retrieving information from electronic health records (EHRs), the use of acronyms (e.g. ‘HIV’) obscures the corresponding phrase (e.g. ‘human immunodeficiency virus’) whose words (e.g. ‘virus’) cannot be indexed by a search engine and, hence, cannot be retrieved. On the other hand, the highly polysemous nature of acronyms (e.g. ‘MRS’ can be interpreted as ‘magnetic resonance spectroscopy’, ‘Melkersson-Rosenthal syndrome’ or a courtesy title prefixed to the name of a married woman) may result in retrieval of irrelevant documents. These problems can be resolved by automatically mapping acronyms to correct interpretation in an external dictionary (e.g. Bodenreider, 2004) based on their context of use. This may be viewed as a word sense disambiguation problem (Agirre and Stevenson, 2006), which is commonly approached by supervised machine learning. Supervised methods are trained using a set of manually annotated examples. Among other factors, the performance of machine learning models and the significance of test results depend on the size of the dataset used for training and testing, respectively. A recent systematic review of clinical text data in machine learning revealed the data annotation bottleneck as one of the key obstacles to machine learning approaches to clinical NLP (Spasić and Nenadić, 2020). The need to preserve patient privacy further narrows this bottleneck by removing crowdsourcing as a viable option for annotation. For synthetic data, crowdsourcing remains an option, but it becomes an expensive commodity due to medical expertise required. We suggest a novel application of existing NLP methods on scientific abstracts to simulate clinical narrative style of acronym usage and annotate them automatically with the correct senses. This, in turn, allows for creation of large datasets that can be used to train supervised approaches to word sense disambiguation of biomedical acronyms.

## 2 System design

**Fig. 1.**
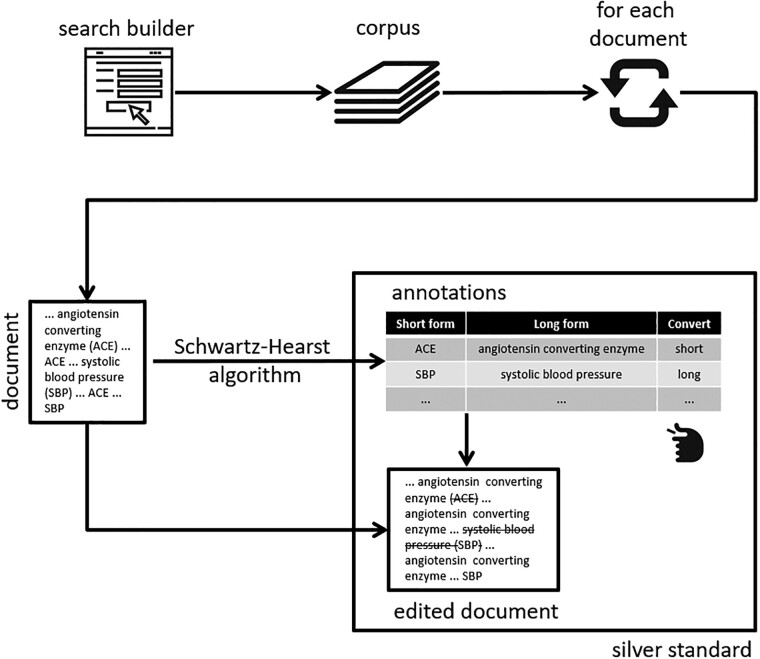
System design

The prevalence of acronyms in biomedical domains (Liu *et al.*, 2002) gave rise to proliferation of methods that extract acronym definitions from text. Most of these methods focus on biomedical literature and have been evaluated on abstracts (e.g. Ao and Takagi, 2005; Chang *et al.*, 2002; Gaudan *et al.*, 2005; Liu and Friedman, 2003; Okazaki and Ananiadou, 2006; Pustejovsky *et al.*, 2001; Schwartz and Hearst, 2003; Sohn *et al.*, 2008; Wren and Garner, 2002; Yu *et al.*, 2003, 2007). They rely on scientific writing conventions, which prescribe that all acronyms need to be defined the first time they are mentioned in a document by specifying the full form followed by the acronym, written within parentheses in uppercase. These conventions are modelled by pattern matching rules to identify potential acronym definitions followed by heuristic alignment of the acronym against its full form. A simple algorithm for identifying acronyms by Schwartz and Hearst (2003), which performs at 96% precision and 82% recall, is by far the most referenced method of its kind. It can be embedded easily into NLP algorithms to support more complex tasks, e.g. multi-word term recognition (Spasić, 2018, 2021). Similarly, it forms the backbone of the system described here (Fig. 1). The Schwartz-Hearst algorithm is used to recognize acronym definitions. As a result, an acronym is linked to its full form, i.e. sense.

To artificially simulate clinical narrative style of acronym usage, either an acronym or its full form needs to be used consistently throughout a single document. Therefore, given a document, the system first choses between an acronym and its full form randomly and then replaces all occurrences of the chosen item by its counterpart. Whenever the full form is removed from text, it is retained as the sense annotation and can, therefore, be used to train machine learning approaches to word sense disambiguation. By repeating this process on many documents, a large training dataset can be created automatically.

## 3 Implementation

The system has been implemented as a web-based application. The main source of data is MEDLINE, a bibliographic database of scientific articles covering the fields relevant to clinical applications including medicine, nursing, pharmacy, dentistry and healthcare (MEDLINE, 2021). MEDLINE is publicly available online and provides free access to abstracts of the articles it indexes. These abstracts are used as free-text documents to assemble a corpus (Fig. 1) whose topical coverage is constrained by the search query. This constraint in turn fosters the ‘one sense per discourse’ hypothesis (Gale *et al.*, 1992). For example, in a corpus retrieved using the search terms ‘knee’ and ‘MRI’, it is reasonable to assume that any mention of the acronym ‘ACL’ would have a single meaning, which would be that of ‘anterior cruciate ligament’. This is in line with expectations from a thematic corpus of clinical narratives. For example, a corpus of knee MRI reports would be expected to uphold a ‘single sense per discourse’ hypothesis.

The search is performed using PubMed (PubMed, 2021), a search engine designed specifically to retrieve information from MEDLINE. A search query, which follows PubMed's syntax to combine field names, MeSH terms, keywords and Boolean operators, is obtained from a user using the front-end of our web-based application. It is then passed onto PubMed using its API to retrieve the corresponding documents. The API limits the number of results returned to 500 per query, so the application may take some time to complete the search in multiple batches. To prevent unnecessary processing and save time, users are given an option to limit the number of results themselves depending on their needs. Once retrieved, all documents are processed in the way described in Figure 1. In addition, all long forms are used to search the Unified Medical Language System (UMLS, 2021) to obtain their unique concept identifier. This helps unify different long forms of the same acronym. For example, if ‘DM2’ is linked to ‘diabetes mellitus type 2’, ‘diabetes mellitus type II’ and ‘type two diabetes mellitus’, then the corresponding sense will be the same as all three long forms have the same concept identifier, C0011860. The UMLS is searched using its own API, which has a rate limit of 20 requests per second per IP address, so the web application may take some time to process all long forms.

All information is managed in a MongoDB database. Once the corpus has been processed, it can be downloaded together with the sense inventory in a simple JSON format ready to be processed locally by other downstream NLP applications.

## 4 Case study

We developed a case study to practically demonstrate how the system can be employed to create a dataset that can be used to train a supervised approach to WSD of biomedical acronyms. To collect abstracts relevant to clinical applications, we created a PubMed query using a keyword ‘clinical’ and a suffix ‘logy’ to refer to various clinical domains (e.g. gastroenterology) while excluding certain keywords (e.g. biology). All abstracts were retrieved and annotated automatically by the system. All non-ambiguous acronyms, i.e. those mapped to a single long form uniquely identified in the UMLS, were discarded leaving a total of 963 ambiguous acronyms with a mean of 4.09 potential long forms per acronym (minimum 2, maximum 27, median 3).

The samples of ambiguous acronyms were created to include a sentence in which an acronym occurred, the acronym itself, a potential long form and a label. A positive label was used to indicate the correct long form. A negative sample was created by taking the same sentence but choosing an incorrect long form from all possible long forms of the given acronym.

For each of the 963 ambiguous acronyms, a total of 1000 samples (or 10% if <10 000 samples were available) were drawn out randomly and reserved for validation and testing, respectively. A total of 16 130 782 remaining samples were retained for training.

Next, the disambiguation of an acronym against the potential long forms was performed by using a transformer-based architecture similar to that of Huang *et al.* (2019). Specifically, we fined-tuned Bidirectional Encoder Representations from Transformers (BERT) (Devlin *et al.*, 2018), which can be used to model the relationship of a pair of texts. In our case, we are modelling a relationship between (i) a sentence that contains an acronym and (ii) the acronym itself together with its potential long form. A binary classifier was then trained to recognize the correct long form. The accuracy, precision, recall and F1 score achieved were 0.946, 0.945, 0.949 and 0.947, respectively. We compared this performance to that of a naïve baseline classifier based on the most frequently occurring long form, which achieved 0.650, 0.692, 0.540 and 0.607, respectively.

This case study provides evidence that our system can be used to provide weak supervision of highly accurate machine learning algorithms for acronym disambiguation. In this case study, we used long form candidates from the UMLS. This is in line with the majority of studies on acronym disambiguation, which use an external dictionary to source potential long forms. Alternatively, an unsupervised method such as FlexiTerm (Spasić, 2021; Spasić *et al.*, 2013) could be used to extract multi-word terms directly from a given corpus and then select potential long forms by applying the Schwartz and Hearst (2003) algorithm to acronym-term pairs.

## 5 Conclusion

We described a web-based application that uses a corpus of scientific abstracts to simulate clinical narrative style of acronym usage and annotate them automatically with the correct senses, which in turn can be used to train supervised approaches to word sense disambiguation of biomedical acronyms. It helps navigate the problems associated with patient privacy and manual annotation overhead associated with the use of clinical text data in machine learning (Spasić and Nenadić, 2020). Even though the application can be used to create of large training datasets automatically, it is relatively slow due to limitations associated with the use of external APIs. However, this is not a major issue as the acquisition of training data is seen as batch processing rather than real-time processing.

*Financial Support*: none declared.

*Conflict of Interest:* none declared.
